# Effect of Prophylactic Incisional Negative Pressure Wound Therapy on Surgical Site Infection After Emergency Laparotomy: An Updated Meta‐Analysis With Trial Sequential Analysis

**DOI:** 10.1002/wjs.70526

**Published:** 2026-08-17

**Authors:** Ana Luíza Rocha Soares Menegat, Brenda Luana Rocha Soares Menegat, Sara Bravin, Jacopo Lin, Henrique Wiederkehr

**Affiliations:** ^1^ University of Caxias do Sul Caxias do Sul Brazil; ^2^ Independent Researcher Italy; ^3^ School of Medicine Vita‐Salute San Raffaele University Milan Italy; ^4^ Faculdades Pequeno Príncipe Curitiba Brazil

**Keywords:** emergency laparotomy, meta‐analysis, negative pressure wound therapy, surgical site infection

## Abstract

**Introduction:**

Patients undergoing emergency laparotomy are at high risk of surgical site infections (SSI), which contribute substantially to postoperative morbidity. Incisional negative pressure wound therapy (iNPWT) has been shown to reduce the incidence of SSI following elective surgery, however, its effectiveness in the emergency setting remains uncertain. Therefore, we conducted a systematic review and meta‐analysis to evaluate the effectiveness of iNPWT in reducing SSI after emergency laparotomy.

**Methods:**

We searched PubMed, Embase, and Cochrane through November 2025 for randomized controlled trials (RCTs) and observational studies comparing iNPWT to standard dressings after emergency laparotomy. Odds ratios (OR) and mean differences (MD) were pooled for relevant outcomes with 95% confidence intervals using random‐effects and inverse variance weighting. A two‐tailed *p*‐value < 0.05 was considered significant. Prespecified subgroup analyses restricted to RCTs were performed. Statistical heterogeneity was assessed using the I^2^ statistic.

**Results:**

Fifteen studies (6 RCTs and 9 observational) including 3369 patients were analyzed. It was found that iNPWT was associated with significantly lower odds of overall (OR 0.36; *p* = 0.0003), superficial, and deep SSI, with consistent findings in RCT‐only analysis (OR 0.41; *p* = 0.0221). iNPWT was also associated with lower odds of seroma (OR 0.57; *p* = 0.0325), with consistent results observed in RCT‐only analyses (OR 0.54; *p* = 0.025). Hospital length of stay was shorter in the iNPWT group (MD −1.49 days; *p* = 0.04), and iNPWT was associated with lower odds of wound dehiscence (OR 0.47; *p* = 0.0062), however, these findings were not confirmed in RCT‐only analyses. Postoperative complications, classified according to the Clavien–Dindo system, did not differ between groups (OR 0.93; *p* = 0.4973). Overall mortality was comparable between groups, although iNPWT was associated with higher follow‐up mortality. This finding was not confirmed in RCTs.

**Conclusion:**

In patients undergoing emergency laparotomy, iNPWT was associated with reduced odds of SSI and wound‐related complications compared with standard dressings. Although iNPWT was associated with higher follow‐up mortality, this finding was not confirmed in RCT‐only analyses.

## Graphical abstract

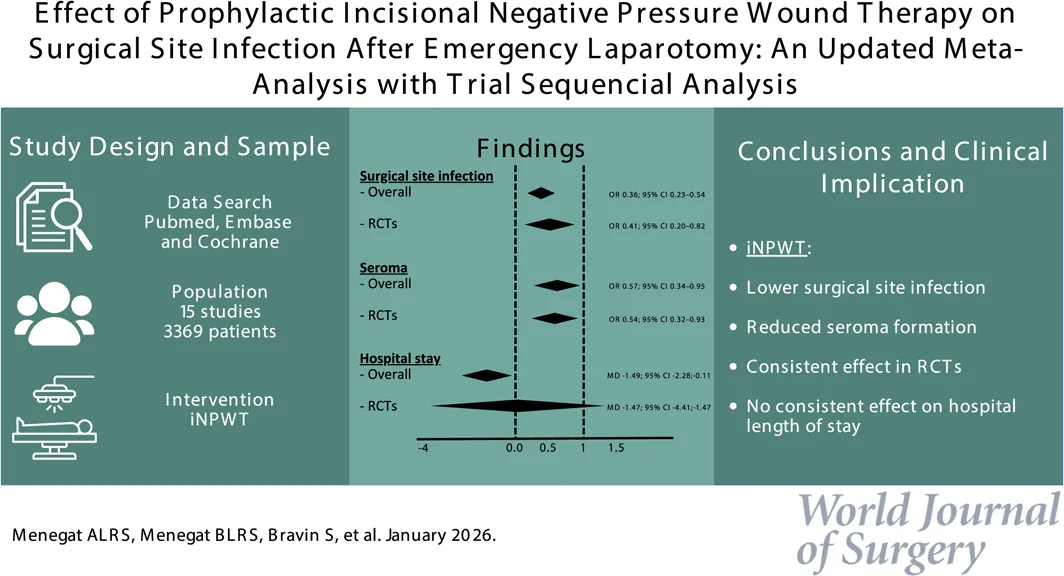

## 1 Introduction

Surgical site infection (SSI) is the most common healthcare‐associated infection following surgery and has a significant impact on patients' morbidity and mortality [1]. The incidence of SSI following emergency laparotomy is estimated to be 25%–40% higher than that observed after elective surgery and increases further in contaminated procedures [2, 3, 4, 5]. The United States Centers for Disease Control and Prevention (CDC) defines an SSI as an infection related to a surgical procedure that occurs near the surgical site within 30 days following a surgery (or up to 90 days following surgery when an implant is involved) [6]. Furthermore, incisional SSIs can be divided into those involving only the skin and subcutaneous tissue (superficial incisional SSI) and those involving deeper layers of the incision (deep incisional SSI).

One strategy to reduce SSI is the prophylactic use of incisional negative pressure wound therapy (iNPWT) applied to closed incisions. iNPWT involves the application of continuous negative pressure, typically ranging from 70 to 125 mmHg, over a primarily closed wound, promoting removal of excess fluid, attenuation of the local inflammatory response within the subcutaneous space, and stimulation of wound healing processes [7]. Robust evidence supports the effectiveness of iNPWT in reducing SSI in elective general surgical procedures, as demonstrated in a recent meta‐analysis [8]. However, the use of iNPWT after emergency laparotomy remains debatable.

Emerging evidence suggests that the use of iNPWT in the emergency setting may reduce SSI rates [5, 9, 10, 11], however, these findings have not been replicated in other studies [12, 13, 14]. Patients undergoing emergency laparotomy are highly heterogeneous and present different risk factors for SSI, such as underlying pathology and operative approach. Currently, several randomized controlled trials (RCTs) are investigating the use of prophylactic iNPWT for closed emergency laparotomy wounds [15, 16].

Although previous meta‐analyses have evaluated the use of iNPWT in both elective and emergency surgical settings, they did not specifically focus on emergency laparotomy or synthesize the evidence from recently published studies [17, 18]. Since the publication of these reviews, additional studies have become available, providing new evidence that warrants an updated synthesis. Therefore, the present study focused exclusively on emergency laparotomy and incorporated these newly published studies, with a subgroup analysis restricted to RCTs and trial sequential analysis (TSA) to further assess the robustness and conclusiveness of the available evidence. This study aims to systematically review and meta‐analyze the available evidence addressing the efficacy of iNPWT in reducing SSI rates and other wound‐related complications among patients undergoing emergency laparotomy.

## 2 Methods

### 2.1 Protocol and Registration

This systematic review and meta‐analysis was conducted according to the Cochrane Handbook for Systematic Reviews of Interventions [19]. The reporting complies with the Preferred Reporting Items for Systematic Reviews and Meta‐Analyses (PRISMA) guidelines, with detailed checklists provided in Supporting Information S1: Tables 1 and 2 [20]. The study protocol was prospectively registered in the International Prospective Register of Systematic Reviews (PROSPERO) under registration number CRD420251180732.

### 2.2 Eligibility Criteria

Studies were considered eligible if they met the following criteria: (1) inclusion of adult patients (≥ 16 years) undergoing emergency laparotomy; (2) use of iNPWT as the intervention; (3) inclusion of a comparator group receiving standard wound dressing; (4) reporting of at least one outcome of interest; and (5) RCTs or observational studies.

Studies were excluded if they met any of the following criteria: (1) studies involving delayed primary closure; (2) case reports or case series; (3) conference abstracts; (4) study protocols; or (5) studies with overlapping patient populations (the study with the larger sample size was retained).

### 2.3 Search Strategy

A systematic search of the literature was performed in PubMed, Embase, and the Cochrane Library from database inception through November 2025. The search strategy combined free‐text terms and controlled vocabulary related to emergency laparotomy (e.g., “emergency laparotom*”) and iNPWT (e.g., “negative pressure wound therapy”), with Boolean operators (AND, OR) applied to structure the search queries (Supporting Information S1: Table 3).

All records retrieved from the electronic searches were imported into Rayyan (Rayyan Systems Inc., Qatar) [21] for automated and manual deduplication. Following removal of duplicates, two reviewers (B.L.R.S.M. and S.B.) independently screened titles and abstracts, with discrepancies resolved through consensus.

### 2.4 Data Extraction

Data extraction was independently conducted by two reviewers (J.L. and H.W.) using a standardized data collection form. Any disagreements were resolved by consultation with the first author, who reviewed the extracted data and made the final determination. The following information was extracted from each eligible study: study identification, study design, follow‐up duration, country of origin, characteristics of the iNPWT, including device type and duration of application, total number of patients, sex distribution, age, CDC wound classification, type of surgical procedure performed, and antibiotic use.

When available, effect estimates derived from adjusted analyses (including propensity score–matched or weighted models) were preferentially extracted.

### 2.5 Quality Assessment

The methodological quality of the included studies was assessed using the Cochrane Risk of Bias 2 (RoB 2) tool and the Risk Of Bias In Nonrandomized Studies of Interventions (ROBINS‐I) [22, 23]. Two reviewers (A.L.R.S.M. and B.L.R.S.M.) independently evaluated the risk of bias of each study, with any disagreements resolved by consensus.

Using the RoB 2 tool, risk of bias was evaluated across the domains of the randomization process, deviations from intended interventions, missing outcome data, outcome measurement, and selection of reported results. Each study was judged as having low risk of bias, some concerns, or high risk of bias. The ROBINS‐I tool assessed bias across the domains of confounding, participant selection, intervention classification, deviations from intended interventions, missing data, outcome measurement, and selection of reported results, with studies categorized as having low, moderate, serious, or critical risk of bias.

### 2.6 Endpoints and Subgroup Analysis

The outcomes of interest were prespecified and included SSI, reported as overall incidence and stratified into superficial, deep, and organ/space infections; length of hospital stay and length of intensive care unit (ICU) stay; wound‐related complications, including dehiscence and seroma; overall postoperative morbidity assessed using the Clavien–Dindo classification and analyzed separately for grades < 3 and ≥ 3; mortality; and 30‐day readmission rate.

For outcomes reported in at least three studies, a predefined subgroup analysis was conducted including only RCTs.

### 2.7 Statistical Analysis

All quantitative analyses used random‐effects models. Binary outcomes were pooled as odds ratios (ORs) and continuous outcomes were synthesized as mean differences (MDs), both with 95% confidence intervals (CIs). Meta‐analyses were performed separately for each outcome using inverse‐variance weighting.

Between‐study heterogeneity was modeled by estimating the variance component (τ^2^) using the restricted maximum likelihood (REML) estimator [19]. The method for CI calculation was defined a priori: Wald‐type CIs were applied when no between‐study heterogeneity was detected (*τ* ^2^ = 0), whereas the Hartung–Knapp–Sidik–Jonkman (HKSJ) approach was employed in the presence of heterogeneity (*τ* ^2^ > 0) provided that at least two studies contributed to the pooled estimate [24]. Statistical heterogeneity was evaluated using Cochran's Q test and quantified with the I^2^ statistic, interpreted as 0%–40% might not be important, 30%–60% may represent moderate heterogeneity, 50%–90% substantial heterogeneity, and 75%–100% considerable heterogeneity. To enhance interpretability, 95% prediction intervals were calculated to estimate the range of treatment effects that may be observed in future clinical settings, incorporating both the pooled effect estimate and the between‐study heterogeneity [25].

All analyses were performed in R software (version 4.4.1) using the meta (v. 8.2‐1) and metafor (v. 4.8‐0) packages, whereas data extraction and management were conducted with the readxl (v. 1.4.5) and openxlsx (v. 4.2.8) packages. A two‐sided *p*‐value < 0.05 was considered statistically significant [26].

### 2.8 Sensitivity Analysis

To examine the stability of the pooled estimates, a leave‐one‐out sensitivity analysis was performed by iteratively rerunning the meta‐analysis with the removal of one study at a time [27]. This approach enabled evaluation of the influence of individual studies on the overall effect size and on between‐study heterogeneity. In addition, Baujat plots were constructed to visually assess the contribution of each study to heterogeneity and its impact on the combined effect estimate, allowing identification of studies exerting a disproportionate influence on the meta‐analytic results [28]. Sensitivity analyses were restricted to outcomes demonstrating at least moderate heterogeneity (I^2^ > 50%).

### 2.9 Assessment of Publication Bias

Potential publication bias was assessed through visual inspection of funnel plots generated for the pooled analyses, with asymmetry interpreted as a possible indication of publication bias, small‐study effects, or selective reporting [29]. Funnel plot asymmetry was formally evaluated using Egger's regression test for continuous outcomes, whereas Harbord's regression test was applied for binary outcomes expressed as odds ratios [30, 31]. Statistically significant results were considered suggestive of small‐study effects. For outcomes supported by a limited number of studies, the interpretation of funnel plots and asymmetry tests should be approached with caution due to reduced statistical reliability.

### 2.10 Trial Sequential Analysis

TSA was performed to assess the robustness of cumulative evidence and control type I and II errors and was applied only to SSI. TSA was conducted using Trial Sequential Analysis Viewer (version 0.9.5.10 Beta; Copenhagen Trial Unit, Center for Clinical Intervention Research, Rigshospitalet, Copenhagen, Denmark) with a random‐effects DerSimonian–Laird model [32, 33, 34]. A two‐sided *α* of 5% and 80% power were prespecified. The required information size was calculated based on the control event rate and the anticipated intervention effect derived from the pooled odds ratio, using a sample‐size information axis. Monitoring boundaries for benefit, harm, and futility were constructed using the O'Brien–Fleming alpha‐spending function.

## 3 Results

### 3.1 Study Selection and Baseline Characteristics

The literature search identified a total of 906 records (Figure 1). After screening, eight new studies met the eligibility criteria and were included in the review. Together with seven studies from a previous version of the review, a total of 15 studies were included in the final analysis, comprising six RCTs and nine observational studies.

**FIGURE 1:**
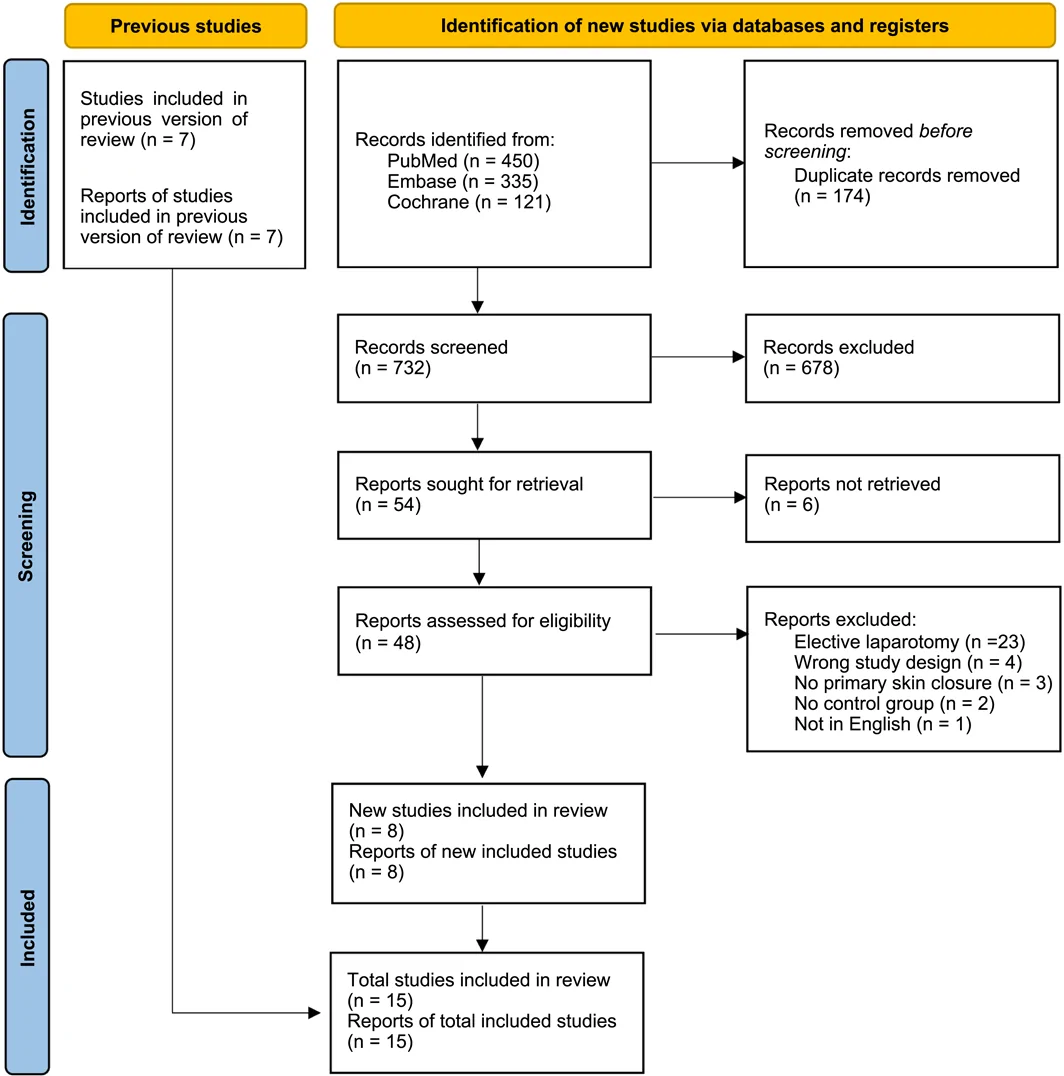
PRISMA flow diagram of study screening and selection.

These studies included 3369 patients undergoing emergency laparotomy, of whom 1712 (50.82%) received iNPWT and 1657 (49.18%) received standard wound dressing [2, 5, 9, 11, 13, 35, 36, 37, 38, 39, 40, 41, 42, 43, 44]. Additional baseline characteristics are summarized in Table 1. Information on CDC wound classification and procedure types is provided in Supporting Information S1: Table 4.

**TABLE 1:** Characteristics of the included studies.

Study	Study design	Follow‐up (days)	Country	iNPWT device	iNPWT duration (days)	Number of patients			Sex ratio (F/M)			Age (years)		Antibiotic use	
						I	C	Total	I	C	Total	I	C	I	C
Atherton, 2025 [35]	RCT	30	UK/Australia	PICO	7 days or until discharge	411	410	821	207/204	224/186	431/390	63.80 ± 15.90	63.70 ± 16.40	Prophylactic 398 Continued postoperatively 234	Prophylactic 401 Continued postoperatively 236
Chung, 2021 [5]	Prospective cohort	30	UK	Prevena	7 days or until discharge	237	237	474	112/125	131/106	243/231	57^b^	60^b^	Preoperative 195	Preoperative 171
Costa, 2024 [36]	RCT	30	Portugal	PICO 41 Prevena 39	7	80	43	123	22/58	16/27	38/85	63.43 ± 17.19	64.14 ± 20.70	NR	NR
Cremonini, 2025 [37]	Prospective case‐control	30	Italy	Prevena	7	37	41	78	16/21	16/25	32/46	70.93 ± 17.73	72.93 ± 13.06	Prophylactic 37 Continued postoperatively 25	Prophylactic 40 Continued postoperatively 23
Garg, 2021 [13]	RCT	30	India	NR	3	25	25	50	13/12	9/16	22/28	46.76 ± 12.20	41.96 ± 8.33	NR	NR
Herczeg, 2025 [38]	RCT	30	Hungary	NR	5	45	45	90	19/26	24/21	43/47	60.96 ± 15.30	67.67 ± 14.30	Prophylactic 20 Therapeutic 24	Prophylactic 17 Therapeutic 21
Kabir, 2023 [11]	Retrospective cohort	30	USA	NR	5	73	79	152	13/60	13/66	26/126	NR	NR	NR	NR
Khan, 2025 [39]	RCT	14	Pakistan	NR	3	30	30	60	12/18	13/17	25/35	42.70 ± 11.40	43.30 ± 12.10	NR	NR
Liu, 2020 [9]	Retrospective cohort	Until wound was healed	Australia	Prevena	5–7	70	70	140	33/37	29/41	62/78	61.30 ± 16.70	63.20 ± 17.10	Preoperative 67 Postoperative 45	Preoperative 68 Postoperative 52
Oishi, 2025 [2]	Retrospective cohort	30	Japan	PICO	3–7	65	62	127	36/29	32/30	68/59	71.23 ± 15.92	70.29 ± 15.17	NR	NR
Philip, 2024 [40]	RCT	30	Malaysia	PICO	7	47	49	96	21/26	22/27	43/53	< 65: 22 > 65: 25	< 65: 23 > 65: 26	Prophylactic or therapeutic 47	Prophylactic or therapeutic 49
Rafaqat, 2024 [41]	Retrospective cohort	30	USA	NR	NR	387	387	774	201/186	208/179	409/365	63.35 ± 15.62	63.00 ± 14.88	NR	NR
Reese, 2025 [42]	Retrospective cohort	Till discharge	USA	Prevena	NR	135	66	201	27/122^a^	12/99^a^	39/221^a^	28^a^ ^,^ ^b^	31^a^ ^,^ ^b^	NR	NR
Schurtz, 2018 [43]	Retrospective case‐control	30	USA	Prevena	4–8	48	48	96	28/20	22/26	50/46	61.40 ± 20.80	61.90 ± 15.10	Preoperative 48	Preoperative 48
Tsukazaki, 2024 [44]	Prospective cohort	30	Japan	NR	10	22	65	87	10/12	33/32	43/44	74.15 ± 12.56	70.54 ± 16.43	NR	NR

Abbreviations: C, control; F, female; I, intervention; iNPWT, incisional negative pressure wound therapy; M, male; NR, not reported; RCT, randomized clinical trial.

^a^ Baseline characteristics were reported for the total study population and not limited to patients undergoing primary skin closure.

^b^ Data reported as mean.

### 3.2 Surgical Site Infection

The pooled analysis showed that iNPWT was associated with lower odds of overall SSI compared with standard dressing (OR 0.36; 95% CI 0.23 to 0.54; *p* = 0.0003; Figure 2a), with substantial heterogeneity (I^2^ = 72.00%). However, the prediction interval crossed the null effect. In RCTs only, iNPWT was also associated with lower odds of SSI (OR 0.41; 95% CI 0.20 to 0.82; *p* = 0.0221; I^2^ = 73.80%; Figure 2b). Subtype analyses showed lower odds of superficial SSI (OR 0.37; 95% CI 0.27 to 0.52; *p* < 0.0001; I^2^ = 0%; Figure 2c) and deep SSI (OR 0.37; 95% CI 0.19 to 0.71; *p* = 0.0027; I^2^ = 0%; Figure 2d), but not in organ/space SSI (OR 0.76; 95% CI 0.50 to 1.17; *p* = 0.2099; I^2^ = 0%; Figure 2e).

**FIGURE 2:**
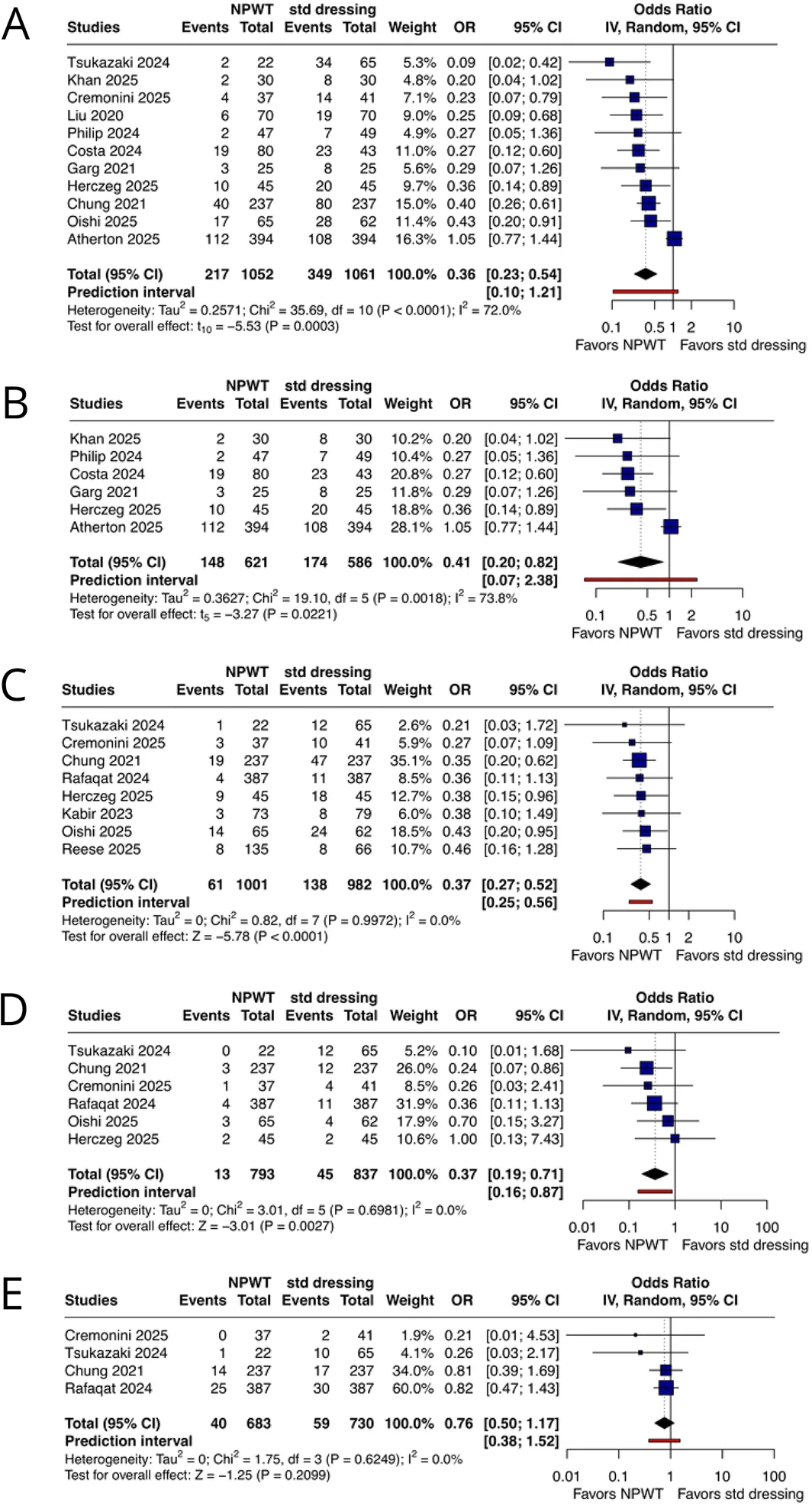
Forest plots comparing iNPWT and standard dressing for surgical site infection outcomes: (a) overall SSI; (b) overall SSI in the subgroup analysis restricted to RCTs; (c) superficial SSI; (d) deep SSI; and (e) organ/space SSI.

### 3.3 Length of Hospital Stay and ICU Stay

The pooled analysis showed that iNPWT was associated with a shorter length of hospital stay compared to standard dressing (MD −1.49 days; 95% CI −2.88 to −0.11; *p* = 0.04; Figure 3a), with considerable heterogeneity (I^2^ = 82.10%). However, the prediction interval crossed the null effect. In RCTs only, no significant difference in hospital stay was observed (MD −1.47 days; 95% CI −4.41 to 1.47; *p* = 0.26; I^2^ = 91.00%; Figure 3b).

**FIGURE 3:**
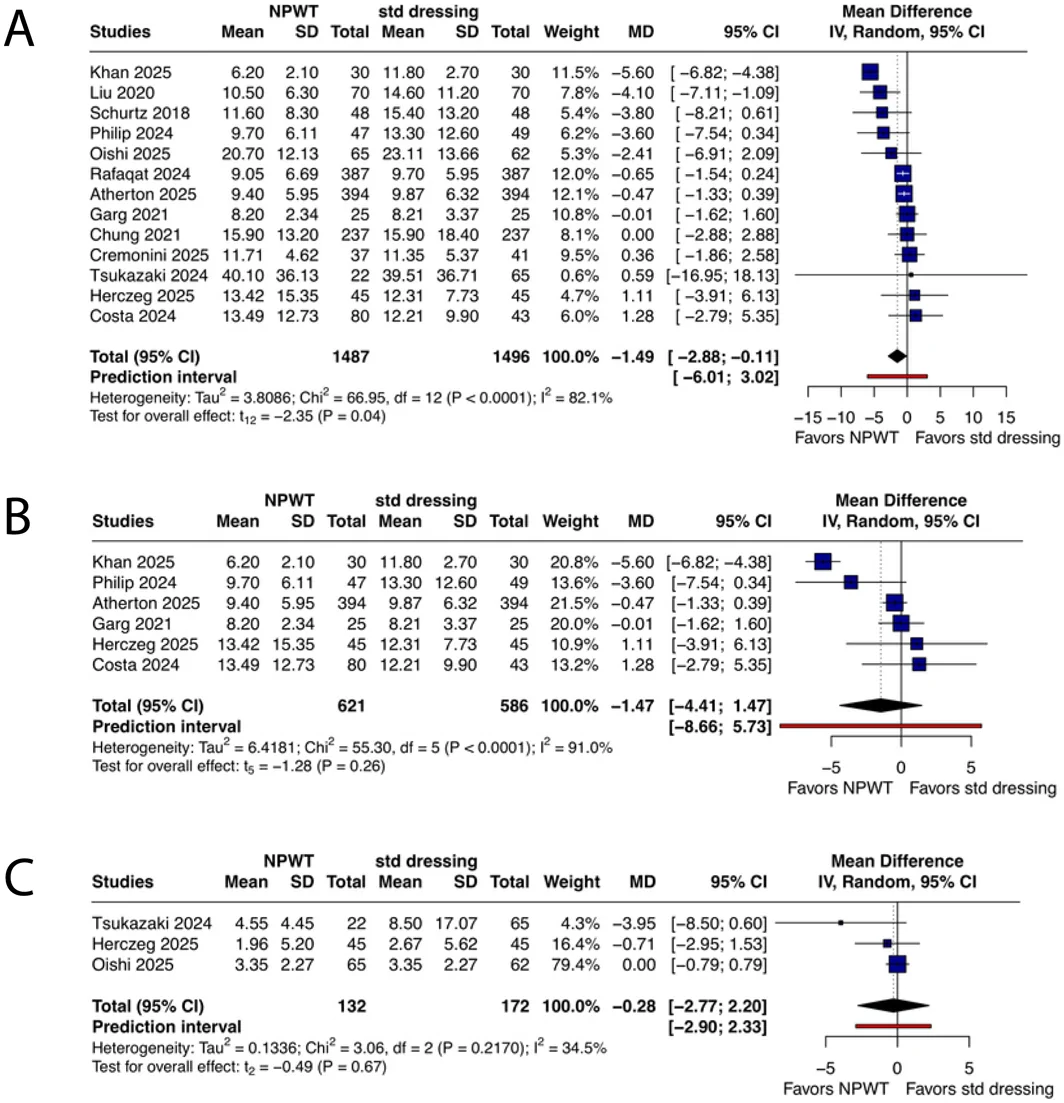
Forest plots comparing iNPWT and standard dressing for length of stay outcomes: (a) hospital length of stay; (b) hospital length of stay in the subgroup analysis restricted to RCTs; and (c) ICU length of stay.

For ICU length of stay, pooled analysis showed no significant difference between groups (MD −0.28 days; 95% CI −2.77 to 2.20; *p* = 0.67; Figure 3c), with low‐to‐moderate heterogeneity (I^2^ = 34.50%). The prediction interval crossed the null effect.

### 3.4 Wound‐Related Complications

iNPWT was associated with lower odds of wound dehiscence compared with standard dressing (OR 0.47; 95% CI 0.29 to 0.75; *p* = 0.0062; Figure 4a), with low‐to‐moderate heterogeneity (I^2^ = 35.50%). The prediction interval crossed the null effect. In RCTs only, no significant difference was observed (OR 0.47; 95% CI 0.19 to 1.16; *p* = 0.0805; I^2^ = 53.10%; Figure 4b).

**FIGURE 4:**
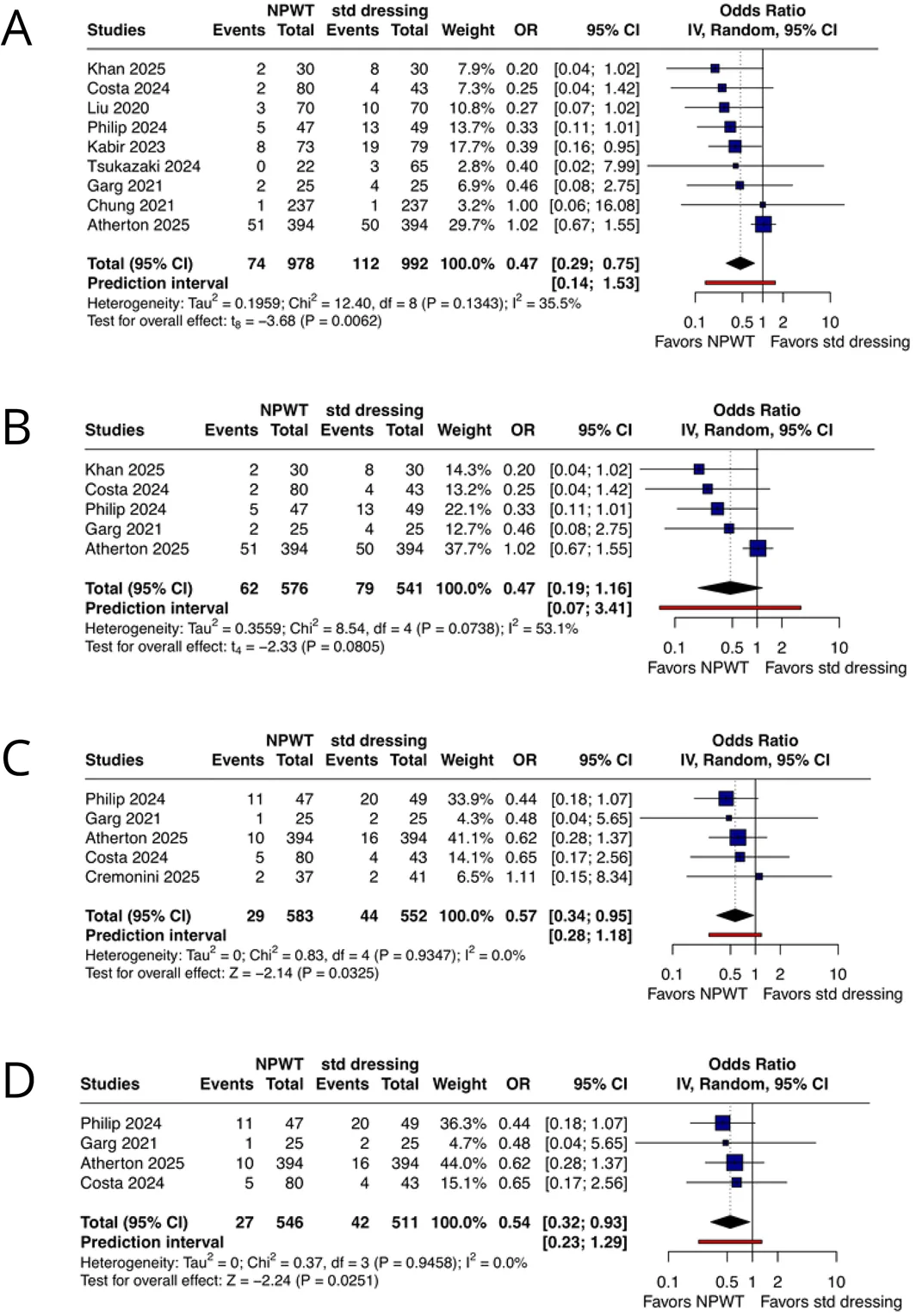
Forest plots comparing iNPWT and standard dressing for wound‐related complications: (a) wound dehiscence; (b) wound dehiscence in the subgroup analysis restricted to RCTs; (c) seroma; and (d) seroma in the subgroup analysis restricted to RCTs.

iNPWT also was associated with lower odds of seroma formation versus standard dressing (OR 0.57; 95% CI 0.34 to 0.95; *p* = 0.0325; I^2^ = 0%; Figure 4c), although the prediction interval crossed the null effect. In RCTs, this reduction remained significant (OR 0.54; 95% CI 0.32 to 0.93; *p* = 0.025; I^2^ = 0%; Figure 4d).

No significant difference in hematoma formation was observed (OR 1.72; 95% CI 0.67 to 4.40; *p* = 0.2578; I^2^ = 0%; Supporting Information S1: Figure 1), with a wide prediction interval crossing the null effect.

### 3.5 Clavien‐Dindo Classification of Complications

The pooled analysis demonstrated no significant difference between iNPWT and standard dressing in overall postoperative complications according to the Clavien–Dindo classification (OR 0.93; 95% CI 0.76 to 1.15; *p* = 0.4973; I^2^ = 0%; Supporting Information S1: Figure 2). The prediction interval crossed the null effect. Stratified analyses also showed no differences for major (Clavien–Dindo ≥ III: OR 0.80; 95% CI 0.55 to 1.16; *p* = 0.2303; I^2^ = 0%) or minor complications (Clavien–Dindo < III: OR 1.00; 95% CI 0.78 to 1.28; *p* = 0.9922; I^2^ = 1.90%), with no subgroup interaction by severity (*p* = 0.3226).

### 3.6 Mortality

The pooled analysis demonstrated no significant difference in overall mortality between iNPWT and standard dressing (OR 1.08; 95% CI 0.72 to 1.60; *p* = 0.6691; I^2^ = 0%; Figure 5). The prediction interval crossed the null effect. Stratified analyses showed no difference in 30‐day mortality (OR 1.02; 95% CI 0.49 to 2.16; *p* = 0.9005), but higher odds of follow‐up mortality with iNPWT, favoring standard dressing (OR 1.66; 95% CI 1.13 to 2.42; *p* = 0.0291). In‐hospital mortality could not be pooled (single study). Subgroup differences were significant (*p* for subgroup differences = 0.0117). In RCTs only, mortality did not differ between groups (OR 1.01; 95% CI 0.28 to 3.72; *p* = 0.9701; I^2^ = 0%; Supporting Information S1: Figure 3), with no subgroup difference (*p* = 0.0569).

**FIGURE 5:**
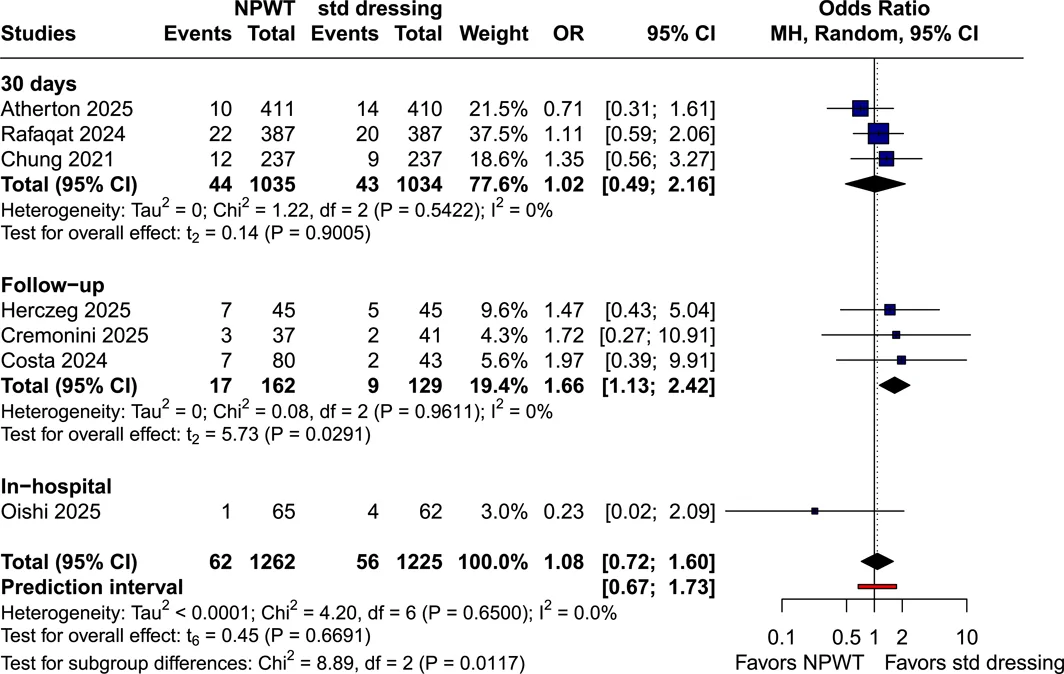
Forest plot comparing iNPWT and standard dressing for mortality outcomes, stratified by follow‐up period: 30‐day mortality, mortality during follow‐up, and in‐hospital mortality.

### 3.7 30‐Day Readmission

The pooled analysis demonstrated no significant difference in 30‐day readmission between iNPWT and standard dressing (OR 0.83; 95% CI 0.39 to 1.73; *p* = 0.4750; Supporting Information S1: Figure 4), with low‐to‐moderate heterogeneity (I^2^ = 29.90%). The prediction interval crossed the null effect.

### 3.8 Quality Assessment

The RCTs assessed using the RoB 2 tool (Supporting Information S1: Figure S5) demonstrated a consistently low risk of bias across all evaluated domains. In contrast, the observational studies evaluated with the ROBINS‐I tool (Supporting Information S1: Figure S6) showed an overall low‐to‐moderate risk of bias, with the main concerns arising from bias due to confounding (D1). All remaining domains were predominantly judged as low risk, resulting in an overall moderate risk of bias for most nonrandomized studies.

### 3.9 Sensitivity Analysis

Sensitivity analyses were performed for overall SSI, SSI restricted to RCTs, length of hospital stay (overall and RCT‐only), and wound dehiscence restricted to RCTs. Exclusion of Atherton et al. [35] eliminated heterogeneity in the analyses of overall SSI, SSI restricted to RCTs, and wound dehiscence restricted to RCTs (I^2^ = 0%), identifying this study as the main source of heterogeneity. For length of hospital stay, exclusion of Khan et al. [39] reduced heterogeneity to 14.40%, with complete resolution in the RCT‐only analysis (I^2^ = 0%). Across all leave‐one‐out analyses, pooled effect estimates remained stable, supporting the robustness of the findings (Supporting Information S1: Figures 7–11).

Baujat analyses were consistent with these results, confirming Atherton et al. [35] and Khan et al. [39] as the principal contributors to heterogeneity and influence in the respective outcomes (Supporting Information S1: Figures 12–16).

### 3.10 Publication Bias

Publication bias was assessed for all outcomes (Supporting Information S1: Figures 17–33). No evidence of small‐study effects was detected for superficial (*p* = 0.7975) or deep SSI (*p* = 0.3354), length of hospital stay (*p* = 0.8604), length of hospital stay restricted to RCTs (*p* = 0.9270), ICU length of stay (*p* = 0.2044), seroma (*p* = 0.4587), seroma restricted to RCTs (*p* = 0.9858), hematoma (*p* = 0.8830), Clavien–Dindo complications (*p* = 0.6658), overall mortality (*p* = 0.9670), mortality restricted to RCTs (*p* = 0.0646), and 30‐day readmission (*p* = 0.9197). In contrast, evidence of funnel plot asymmetry was observed for overall SSI (*p* = 0.0038), SSI restricted to RCTs (*p* = 0.0113), organ/space SSI (*p* = 0.0216), wound dehiscence (*p* = 0.0247), and wound dehiscence restricted to RCTs (*p* = 0.0273).

### 3.11 Trial Sequential Analysis

TSA for SSI demonstrated that the cumulative Z‐curve crossed both the conventional boundary for statistical significance and the trial sequential monitoring boundary for benefit, indicating firm evidence in favor of iNPWT. Although the required information size (RIS = 2255 patients) was not fully reached, the Z‐curve remained above the benefit boundary at the final accrued information size (2113 patients), suggesting that the observed reduction in SSI is unlikely to be due to random error (Supporting Information S1: Figure 34).

## 4 Discussion

In this meta‐analysis of 15 studies including 3369 patients undergoing emergency laparotomy, we evaluated the impact of prophylactic iNPWT on postoperative outcomes. The main findings were as follows: (1) lower odds of overall SSI associated with iNPWT, including superficial and deep SSI, with consistent results observed in RCTs, whereas no significant difference was found for organ/space SSI; (2) lower odds of seroma formation associated with iNPWT, with this finding remaining consistent when the analysis was restricted to RCTs; (3) a shorter length of hospital stay associated with iNPWT in the overall analysis, which was not confirmed in RCT‐only analyses, with no difference in ICU length of stay; and (4) no significant differences were observed in other evaluated perioperative outcomes, whereas higher odds of follow‐up mortality were observed in the iNPWT group.

The use of iNPWT on primarily closed surgical incisions has been increasingly adopted in high‐risk surgical settings, including emergency laparotomy, based on accumulating evidence from RCTs and international consensus documents. Clinical studies in abdominal and colorectal surgery have demonstrated that prophylactic iNPWT applied to closed incisions is associated with lower rates of SSI, particularly in high‐risk patients. These include those with contaminated or potentially contaminated fields, sepsis, obesity, or other risk factors for impaired wound healing (6.50% vs. 15.10%, *p* = 0.05 [Curran et al. 45]; 9% vs. 19%, *p* = 0.02 [Abadía et al. [46]]). Based on these data, the World Health Organization issued a conditional recommendation for the use of iNPWT on closed surgical incisions in high‐risk wounds as part of strategies to prevent SSI. However, this recommendation was explicitly graded as being supported by low‐quality of evidence, reflecting heterogeneity across studies and remaining uncertain regarding the magnitude of benefit [47].

In this context, our meta‐analysis in patients undergoing emergency laparotomy demonstrated significantly lower odds of SSI associated with iNPWT compared with standard dressings. This effect remained consistent when the analysis was restricted to RCTs, supporting the validity of the findings based on higher‐level evidence. TSA further supported the robustness of this effect, as the cumulative Z‐curve crossed the conventional boundary for benefit, with only a small number of additional patients required to reach the predefined information size. From a mechanistic perspective, iNPWT may reduce SSI risk by facilitating continuous evacuation of wound exudate and limiting subcutaneous fluid accumulation. This may decrease seroma formation, stabilizing wound edges, and reducing lateral tension and micromotion across the incision [48]. Additionally, improvements in local perfusion and reductions in interstitial edema may create a wound environment more conducive to primary healing, making it less susceptible to infection [49].

Most available meta‐analyses evaluating prophylactic iNPWT after laparotomy have included heterogeneous populations, combining both elective and emergency procedures rather than focusing exclusively on emergency surgery. A meta‐analysis of RCTs including elective and emergency procedures demonstrated a significant protective effect of iNPWT against SSI (RR 0.665; *p* = 0.0098) [17]. Similarly, a large systematic review and meta‐analysis of general abdominal surgery pooled elective and emergency procedures, as well as randomized and nonrandomized studies. This analysis reported a significantly lower risk of SSI with iNPWT compared with standard dressings in the overall analysis (RR 0.57; *p* < 0.001); however, this benefit was no longer statistically significant when restricted to RCTs (RR 0.64; *p* = 0.07) [18]. Importantly, these previous meta‐analyses did not demonstrate consistent benefits of iNPWT on secondary outcomes such as seroma formation or length of hospital stay, which were either inconsistently reported or not statistically significant. In contrast, the present meta‐analysis, restricted exclusively to emergency laparotomy, with subgroup analyses limited to RCTs and complemented by TSA, demonstrated a significant reduction in seroma as well as a shorter length of hospital stay. However, when analyses were restricted to RCTs, the reduction in length of hospital stay was no longer statistically significant.

A signal toward higher mortality during follow‐up was observed in the iNPWT group, however, this finding should be interpreted with caution. Given the local nature of the intervention, a direct causal relationship appears unlikely. In the overall analysis, a significant subgroup difference was observed between 30‐day and follow‐up mortality (*p* for subgroup differences = 0.0117), driven by the absence of an effect on 30‐day mortality and higher odds of mortality during follow‐up. However, this subgroup difference was not confirmed in analyses restricted to RCTs (*p* for subgroup differences = 0.0569), suggesting that the observed association may have been influenced by residual confounding, differences in baseline patient characteristics, or other biases inherent to observational studies. Furthermore, interpretation of mortality outcomes is limited by inconsistent reporting across studies. In‐hospital mortality, for example, was reported in only one study and therefore could not be pooled, precluding robust conclusions regarding early postoperative mortality.

The duration of iNPWT application varied considerably across studies. Although some trials applied a standardized short course of 3 days (Garg et al.), others extended therapy up to 10 days (Tsukazaki et al.) or used variable durations based on clinical evolution (Oishi et al.) [2, 13, 44]. Differences were also observed in dressing change intervals, with some studies reporting changes every 24 hours in conventional dressings versus every 72 hours with iNPWT, as reported by Khan et al. [39]. Given that the proposed mechanisms of iNPWT, including exudate removal, edema reduction, and wound stabilization, are time‐dependent, variability in treatment duration constitutes a source of methodological heterogeneity across studies.

Another potential source of heterogeneity relates to differences in the negative pressure settings used across studies, ranging from −80 mmHg [35, 40] to −125 mmHg [5, 9, 37, 39, 42], whereas Tsukazaki et al. used −120 mmHg and Herczeg et al. applied either −90 or −120 mmHg depending on wound output [38, 44]. This variation may be clinically relevant, as experimental evidence suggests that the mechanical effects NPWT are pressure‐dependent, with increasing wound contraction and wound edge forces observed up to approximately −120 mmHg. Notably, a pressure of −80 mmHg achieves nearly 85% of the maximal mechanical effect observed at −120 mmHg, whereas substantially lower pressures (−40 mmHg) may attenuate these effects [50]. Therefore, differences in pressure settings may have influenced treatment efficacy and contributed to heterogeneity across studies.

Significant heterogeneity was also observed in some outcomes, which may be partially explained by differences in the clinical profiles of the included populations across studies. For instance, in the analysis of SSI, the study by Tsukazaki et al. was the only one to exclusively include patients classified as having dirty wounds and demonstrated one of the largest effect sizes favoring iNPWT [44]. Similarly, in the analysis of length of hospital stay, the study by Khan et al. was the only one to exclusively include patients with peritonitis and showed a more pronounced reduction associated with iNPWT, in addition to being the main contributor to heterogeneity in this outcome [39]. These differences likely reflect variation in baseline disease severity and surgical indications across studies, contributing to the observed heterogeneity.

This meta‐analysis has some limitations that should be acknowledged. First, although RCTs were included, a substantial proportion of the evidence was derived from observational studies. This may introduce selection bias and residual confounding. To mitigate this limitation, subgroup analyses restricted to RCTs were performed whenever three or more trials were available. Second, significant heterogeneity was observed in some outcomes, likely reflecting heterogeneous indications for emergency laparotomy across the included studies. Subgroup analyses stratified by indication were not possible. Additionally, the definition of emergency surgery was not standardized among studies, which may have further contributed to clinical heterogeneity. Notably, heterogeneity for SSI was reduced to 0% when infections were analyzed separately as superficial, deep, and organ/space, suggesting that outcome stratification influenced consistency across studies. Third, standard dressing protocols in the control groups were not consistently reported, limiting comparisons across studies and introducing additional methodological heterogeneity. Finally, we were unable to stratify analyses according to the CDC wound classification, which defines baseline SSI risk. Additionally, meta‐regression analyses for SSI were not feasible, as fewer than 10 studies reported these variables.

Future research should aim to standardize technical parameters such as negative pressure settings, dressing type, and optimal duration of therapy. It should also evaluate subpopulations that may derive the greatest benefit, such as patients with high contamination class or specific comorbidity profiles. Additionally, integration of health‐economic evaluations, including cost‐effectiveness and resource utilization, will be important to support guideline recommendations and implementation in varied healthcare settings. Finally, adjunctive strategies integrated with iNPWT may further optimize wound outcomes and broaden its clinical applicability in emergency surgical care.

## 5 Conclusion

In this meta‐analysis of patients undergoing emergency laparotomy, subgroup analyses restricted to RCTs and TSA were performed. Prophylactic iNPWT was associated with a significant reduction in overall SSI, including superficial and deep SSI, as well as seroma, with consistent findings observed in RCT‐only analyses, strengthening the level of evidence supporting this association. Although higher odds of follow‐up mortality were observed in the overall analysis, this finding was not confirmed in the RCT‐only analysis. Overall, these results indicate that iNPWT may be a favorable preventive strategy for SSI in this high‐risk population and support its consideration as part of postoperative wound management following emergency laparotomy.

## Author Contributions

**Ana Luíza Rocha Soares Menegat:** conceptualization, software development, formal analysis, visualization, project administration, writing – original draft, writing – review and editing. **Brenda Luana Rocha Soares Menegat:** software development, formal analysis, visualization, project administration, writing – original draft. **Sara Bravin:** investigation, writing – original draft. **Jacopo Lin:** data curation, writing – original draft. **Henrique Wiederkehr:** data curation, supervision, project administration, writing – original draft, writing – review and editing.

## Funding

The authors have nothing to report.

## Conflicts of Interest

The authors declare no conflicts of interest.

## Supporting information


Supporting Information S1

## Data Availability

The data supporting the findings of this study are available within the article and its supplementary materials and were extracted from previously published studies.
