# Supplementary material for: Effect of Prophylactic Incisional Negative Pressure Wound Therapy on Surgical Site Infection After Emergency Laparotomy: An Updated Meta‐Analysis With Trial Sequential Analysis
Source: World J Surg. 2026 Aug 17;50(10):2848–60. doi: 10.1002/wjs.70526 (PMC13662496; doi:10.1002/wjs.70526)
Supplement: Supplementary file 1 [file WJS-50-2848-s001.docx]

**Effect of Prophylactic Incisional Negative Pressure Wound Therapy on Surgical Site Infection After Emergency Laparotomy: An Updated Meta-Analysis with Trial Sequential Analysis**

***World Journal of Surgery***

Ana Luíza Rocha Soares Menegat^1^, Brenda Luana Rocha Soares Menegat^1^, Sara Bravin M.D.^2^, Jacopo Lin^3^, Henrique Wiederkehr M.D.^4^

^1^University of Caxias do Sul, Caxias do Sul, Brazil

^2^Independent researcher, Italy

^3^School of Medicine, Vita-Salute San Raffaele University, Milan, Italy

^4^Faculdades Pequeno Príncipe, Curitiba, Brazil

**Address for correspondence:** 1130, Francisco Getúlio Vargas Street, Caxias do Sul, 95070-560, Brazil

**Corresponding author:** Ana Luíza Rocha Soares Menegat; analrsmenegat@gmail.com; University of Caxias do Sul, Caxias do Sul, Brazil

Ana Luíza Rocha Soares Menegat ORCID: 0009-0005-9126-0821

Brenda Luana Rocha Soares Menegat ORCID: 0009-0002-5703-1687

Sara Bravin ORCID: 0009-0004-6562-4823

Jacopo Lin ORCID: 0009-0001-6476-0645

Henrique Wiederkehr ORCID: 0000-0002-6357-8286

**Supplementary Material**

**Supplementary Table 1.** PRISMA 2020 Checklist

**Supplementary Table 2.** PRISMA 2020 for Abstract Checklist

**Supplementary Table 3.** Search Strategies

**Supplementary Table 4.** CDC wound classification and types of surgical procedures

**Supplementary Figure 1.** Forest plot comparing NPWT and standard dressing for hematoma

**Supplementary Figure 2.** Forest plot comparing iNPWT and standard dressing for postoperative complications according to the Clavien–Dindo classification, including overall complications and subgroup analyses by complication severity (Clavien–Dindo ≥ III and < III)

**Supplementary Figure 3.** Forest plot comparing NPWT and standard dressing for mortality in the subgroup analysis restricted to RCTs

**Supplementary Figure 4.** Forest plot comparing NPWT and standard dressing for 30-day readmission

**Supplementary Figure 5.** Risk-of-Bias for RCTs performed by Rob-2

**Supplementary Figure 6.** Risk-of-Bias for observational studies by Robins-I

**Supplementary Figure 7.** Leave-one-out sensitivity analysis for SSI

**Supplementary Figure 8.** Leave-one-out sensitivity analysis for SSI in the subgroup analysis restricted to RCTs

**Supplementary Figure 9.** Leave-one-out sensitivity analysis for length of hospital stay

**Supplementary Figure 10.** Leave-one-out sensitivity analysis for length of hospital stay in the subgroup analysis restricted to RCTs

**Supplementary Figure 11.** Leave-one-out sensitivity analysis for wound dehiscence in the subgroup analysis restricted to RCTs

**Supplementary Figure 12.** Baujat plot for SSI

**Supplementary Figure 13.** Baujat plot for SSI in the subgroup analysis restricted to RCTs

**Supplementary Figure 14.** Baujat plot for length of hospital stay

**Supplementary Figure 15.** Baujat plot for length of hospital stay in the subgroup analysis restricted to RCTs

**Supplementary Figure 16.** Baujat plot for wound dehiscence in the subgroup analysis restricted to RCTs

**Supplementary Figure 17.** Funnel plot for SSI

**Supplementary Figure 18.** Funnel plot for SSI in the subgroup analysis restricted to RCTs

**Supplementary Figure 19.** Funnel plot for superficial SSI

**Supplementary Figure 20.** Funnel plot for deep SSI

**Supplementary Figure 21.** Funnel plot for organ/space SSI

**Supplementary Figure 22.** Funnel plot for length of hospital stay

**Supplementary Figure 23.** Funnel plot for length of hospital stay in the subgroup analysis restricted to RCTs

**Supplementary Figure 24.** Funnel plot for ICU length of stay

**Supplementary Figure 25.** Funnel plot for wound dehiscence

**Supplementary Figure 26.** Funnel plot for wound dehiscence in the subgroup analysis restricted to RCTs

**Supplementary Figure 27.** Funnel plot for seroma

**Supplementary Figure 28.** Funnel plot for seroma in the subgroup analysis restricted to RCTs

**Supplementary Figure 29.** Funnel plot for hematoma

**Supplementary Figure 30.** Funnel plot for Clavien-Dindo classification

**Supplementary Figure 31.** Funnel plot for mortality

**Supplementary Figure 32.** Funnel plot for mortality in the subgroup analysis restricted to RCTs

**Supplementary Figure 33.** Funnel plot for 30-day readmission

**Supplementary Figure 34.** Trial sequential analysis comparing NPWT with standard dressing for SSI

**Supplementary Table 1.** PRISMA 2020 Checklist

| **Section and Topic** | **Item #** | **Checklist item** | **Location where item is reported** |
| --- | --- | --- | --- |
| **TITLE** | | |  |
| Title | 1 | Identify the report as a systematic review. | Title page |
| **ABSTRACT** | | |  |
| Abstract | 2 | See the PRISMA 2020 for Abstracts checklist. | Supplementary Table 2 |
| **INTRODUCTION** | | |  |
| Rationale | 3 | Describe the rationale for the review in the context of existing knowledge. | Page 2 |
| Objectives | 4 | Provide an explicit statement of the objective(s) or question(s) the review addresses. | Page 2 |
| **METHODS** | | |  |
| Eligibility criteria | 5 | Specify the inclusion and exclusion criteria for the review and how studies were grouped for the syntheses. | Page 3 |
| Information sources | 6 | Specify all databases, registers, websites, organisations, reference lists and other sources searched or consulted to identify studies. Specify the date when each source was last searched or consulted. | Pages 3-4 |
| Search strategy | 7 | Present the full search strategies for all databases, registers and websites, including any filters and limits used. | Pages 3-4  Supplementary Table 3 |
| Selection process | 8 | Specify the methods used to decide whether a study met the inclusion criteria of the review, including how many reviewers screened each record and each report retrieved, whether they worked independently, and if applicable, details of automation tools used in the process. | Pages 3-4 |
| Data collection process | 9 | Specify the methods used to collect data from reports, including how many reviewers collected data from each report, whether they worked independently, any processes for obtaining or confirming data from study investigators, and if applicable, details of automation tools used in the process. | Page 4 |
| Data items | 10a | List and define all outcomes for which data were sought. Specify whether all results that were compatible with each outcome domain in each study were sought (e.g. for all measures, time points, analyses), and if not, the methods used to decide which results to collect. | Page NO |
|  | 10b | List and define all other variables for which data were sought (e.g. participant and intervention characteristics, funding sources). Describe any assumptions made about any missing or unclear information. | Pages 5-6 |
| Study risk of bias assessment | 11 | Specify the methods used to assess risk of bias in the included studies, including details of the tool(s) used, how many reviewers assessed each study and whether they worked independently, and if applicable, details of automation tools used in the process. | Pages 4-5 |
| Effect measures | 12 | Specify for each outcome the effect measure(s) (e.g. risk ratio, mean difference) used in the synthesis or presentation of results. | Pages 6-7 |
| Synthesis methods | 13a | Describe the processes used to decide which studies were eligible for each synthesis (e.g. tabulating the study intervention characteristics and comparing against the planned groups for each synthesis (item #5)). | Page NO |
|  | 13b | Describe any methods required to prepare the data for presentation or synthesis, such as handling of missing summary statistics, or data conversions. | Pages 6-7 |
|  | 13c | Describe any methods used to tabulate or visually display results of individual studies and syntheses. | Page 4 |
|  | 13d | Describe any methods used to synthesize results and provide a rationale for the choice(s). If meta-analysis was performed, describe the model(s), method(s) to identify the presence and extent of statistical heterogeneity, and software package(s) used. | Pages 6-7 |
|  | 13e | Describe any methods used to explore possible causes of heterogeneity among study results (e.g. subgroup analysis, meta-regression). | Page 6 |
|  | 13f | Describe any sensitivity analyses conducted to assess robustness of the synthesized results. | Page 6 |
| Reporting bias assessment | 14 | Describe any methods used to assess risk of bias due to missing results in a synthesis (arising from reporting biases). | Pages 4-5 |
| Certainty assessment | 15 | Describe any methods used to assess certainty (or confidence) in the body of evidence for an outcome. | Pages 4-6 |
| **RESULTS** | | |  |
| Study selection | 16a | Describe the results of the search and selection process, from the number of records identified in the search to the number of studies included in the review, ideally using a flow diagram. | Figure 1  Pages 7-8 |
|  | 16b | Cite studies that might appear to meet the inclusion criteria, but which were excluded, and explain why they were excluded. | Page NO |
| Study characteristics | 17 | Cite each included study and present its characteristics. | Table 1 |
| Risk of bias in studies | 18 | Present assessments of risk of bias for each included study. | Supplementary Figures 4-5  Page 11 |
| Results of individual studies | 19 | For all outcomes, present, for each study: (a) summary statistics for each group (where appropriate) and (b) an effect estimate and its precision (e.g. confidence/credible interval), ideally using structured tables or plots. | Pages 7-11 |
| Results of syntheses | 20a | For each synthesis, briefly summarise the characteristics and risk of bias among contributing studies. | Page 11 |
|  | 20b | Present results of all statistical syntheses conducted. If meta-analysis was done, present for each the summary estimate and its precision (e.g. confidence/credible interval) and measures of statistical heterogeneity. If comparing groups, describe the direction of the effect. | Pages 7-11 |
|  | 20c | Present results of all investigations of possible causes of heterogeneity among study results. | Pages 11-12 |
|  | 20d | Present results of all sensitivity analyses conducted to assess the robustness of the synthesized results. | Pages 11-12 |
| Reporting biases | 21 | Present assessments of risk of bias due to missing results (arising from reporting biases) for each synthesis assessed. | Page 11 |
| Certainty of evidence | 22 | Present assessments of certainty (or confidence) in the body of evidence for each outcome assessed. | Page NO |
| **DISCUSSION** | | |  |
| Discussion | 23a | Provide a general interpretation of the results in the context of other evidence. | Pages 13-15 |
|  | 23b | Discuss any limitations of the evidence included in the review. | Page 15 |
|  | 23c | Discuss any limitations of the review processes used. | Page 15 |
|  | 23d | Discuss implications of the results for practice, policy, and future research. | Page 15 |
| **OTHER INFORMATION** | | |  |
| Registration and protocol | 24a | Provide registration information for the review, including register name and registration number, or state that the review was not registered. | Page 3 |
|  | 24b | Indicate where the review protocol can be accessed, or state that a protocol was not prepared. | Page NO |
|  | 24c | Describe and explain any amendments to information provided at registration or in the protocol. | Page NO |
| Support | 25 | Describe sources of financial or non-financial support for the review, and the role of the funders or sponsors in the review. | Title page |
| Competing interests | 26 | Declare any competing interests of review authors. | Title page |
| Availability of data, code and other materials | 27 | Report which of the following are publicly available and where they can be found: template data collection forms; data extracted from included studies; data used for all analyses; analytic code; any other materials used in the review. | Page NO |

**Supplementary Table 2.** PRISMA 2020 for Abstract Checklist

| **Section and Topic** | **Item #** | **Checklist item** | **Reported (Yes/No)** |
| --- | --- | --- | --- |
| **TITLE** | | |  |
| Title | 1 | Identify the report as a systematic review. | Yes |
| **BACKGROUND** | | |  |
| Objectives | 2 | Provide an explicit statement of the main objective(s) or question(s) the review addresses. | Yes |
| **METHODS** | | |  |
| Eligibility criteria | 3 | Specify the inclusion and exclusion criteria for the review. | No |
| Information sources | 4 | Specify the information sources (e.g. databases, registers) used to identify studies and the date when each was last searched. | Yes |
| Risk of bias | 5 | Specify the methods used to assess risk of bias in the included studies. | No |
| Synthesis of results | 6 | Specify the methods used to present and synthesise results. | Yes |
| **RESULTS** | | |  |
| Included studies | 7 | Give the total number of included studies and participants and summarise relevant characteristics of studies. | Yes |
| Synthesis of results | 8 | Present results for main outcomes, preferably indicating the number of included studies and participants for each. If meta-analysis was done, report the summary estimate and confidence/credible interval. If comparing groups, indicate the direction of the effect (i.e. which group is favoured). | Yes |
| **DISCUSSION** | | |  |
| Limitations of evidence | 9 | Provide a brief summary of the limitations of the evidence included in the review (e.g. study risk of bias, inconsistency and imprecision). | No |
| Interpretation | 10 | Provide a general interpretation of the results and important implications. | Yes |
| **OTHER** | | |  |
| Funding | 11 | Specify the primary source of funding for the review. | No |
| Registration | 12 | Provide the register name and registration number. | No |

**Supplementary Table 3.** Search Strategies

| **Database** | **Search Strategy** |
| --- | --- |
| **PubMed** | (“Emergency laparotom*” OR Laparotom* OR "Laparotomy" [Mesh] OR “Abdominal surgery” OR “Abdominal incision”) AND (“Negative pressure wound therapy” OR “Negative-pressure wound therapy” OR "Negative-Pressure Wound Therapy" [Mesh] OR NPWP OR iNPWT OR “Closed-incision negative pressure therapy” OR “Closed incision negative pressure therapy” OR “Vacuum therapy” OR "Negative pressure therapy”) |
| **Embase** | ('emergency laparotom*' OR 'laparotom*' OR 'laparotomy'/exp OR 'abdominal surgery' OR 'abdominal incision') AND ('negative pressure wound therapy' OR 'negative-pressure wound therapy' OR 'vacuum assisted closure'/exp OR 'npwp' OR 'inpwt' OR 'closed-incision negative pressure therapy' OR 'closed incision negative pressure therapy' OR 'vacuum therapy' OR 'negative pressure therapy') AND ('wound dressing' OR 'conventional dressing' OR 'standard primary closure' OR 'standard dressing' OR 'standard wound dressing' OR 'conventional wound care' OR 'conventional closure' OR 'dressing') |
| **Cochrane** | ('emergency laparotom*' OR 'laparotom*' OR 'laparotomy' OR 'abdominal surgery' OR 'abdominal incision') AND ('negative pressure wound therapy' OR 'negative-pressure wound therapy' OR 'vacuum assisted closure' OR 'npwp' OR 'inpwt' OR 'closed-incision negative pressure therapy' OR 'closed incision negative pressure therapy' OR 'vacuum therapy' OR 'negative pressure therapy') AND ('wound dressing' OR 'conventional dressing' OR 'standard primary closure' OR 'standard dressing' OR 'standard wound dressing' OR 'conventional wound care' OR 'conventional closure' OR 'dressing') |

**Supplementary Table 4.** CDC wound classification and types of surgical procedures

| *Study* | *CDC Wound Classification*  *n (%)* | | *Procedure Types*  *n (%)* | |
| --- | --- | --- | --- | --- |
|  | *I* | *C* | *I* | *C* |
| Atherton, 2025 | Clean 98 (23.80)  Clean-contaminated 175 (42.60)  Contaminated 81 (19.70)  Dirty 57 (13.90) | Clean 99 (24.10)  Clean-contaminated 176 (42.90)  Contaminated 79 (19.30)  Dirty 56 (13.70) | Bowel (colonic) 202 (49.10)  Bowel (noncolonic) 97 (23.60)  NonBowel 112 (27.30) | Bowel (colonic) 198 (48.30)  Bowel (noncolonic) 99 (24.10)  Nonbowel 112 (27.30)  Other 1 (0.20) |
| Chung, 2021 | NR | NR | Upper GI 21 (9.30)  Colorectal 117 (51.80)  Small bowel 60 (26.50)  Stoma 7 (3.10)  Other 32 (13.50) | Upper GI 26 (11.90)  Colorectal 76 (34.70)  Small Bowel 81 (37.00)  Stoma 10 (4.60)  Other 44 (18.50) |
| Costa, 2024 | Clean 19 (23.75)  Clean-contaminated 19 (23.75)  Contaminated 24 (30)  Infected 18 (22.50) | Clean 10 (23.30)  Clean-contaminated 16 (37.20)  Contaminated 5 (11.60)  Infected 12 (27.90) | Intestinal Occlusion 41 (51.25)  Ischemic emergencies 6 (7.50)  Hernia 5 (6.25)  Trauma 7 (8.75)  Sepsis 19 (23.75)  GI bleeding 2 (2.50) | Intestinal Occlusion 19 (44.20)  Ischemic emergencies 2 (4.70)  Hernia 5 (11.60)  Trauma 3 (7.00)  Sepsis 13 (30.20)  GI bleeding 1 (2.30) |
| Cremonini, 2025 | Contaminated 18 (48.60)  Dirty 19 (51.40) | Contaminated 22 (53.70)  Dirty 19 (46.30) | NR | NR |
| Garg, 2021 | NR | NR | NR | NR |
| Herczeg, 2025 | Clean 1 (2.20)  Clean-contaminated 3 (6.70)  Contaminated 26 (57.80)  Infected 15 (33.30) | Clean 0  Clean-contaminated 7 (15.60)  Contaminated 21 (46.70)  Infected 17 (37.80) | NR | NR |
| Kabir, 2023 | NR | NR | Trauma:  Penetrating 48 (66.00)  Blunt 25 (34.00) | Trauma:  Penetrating 45 (57.00)  Blunt 34 (43.00) |
| Khan, 2025 | NR | NR | Peritonitis | Peritonitis |
| Liu, 2020 | Clean 2 (2.90)  Clean-contaminated 36 (51.40)  Contaminated 15 (21.40)  Dirty 17 (24.30) | Clean 1 (1.40)  Clean-contaminated 37 (52.90)  Contaminated 21 (30)  Dirty 11 (15.70) | Small bowel obstruction from adhesions 9 (12.90%)  Large bowel obstruction from cancer 15 (21.40%)  Small bowel obstruction from hernias 6 (8.60%)  Complicated diverticulitis 9 (12.90%)  Perfurated viscus 2 (2.90%)  Small bowel obstuction from cancer 5 (7.10%)  Anastomotic leak 6 (8.60%)  Others 18 (25.70%) | Small bowel obstruction from adhesions 10 (14.30%)  Large bowel obstruction from cancer 8 (11.40%)  Small bowel obstruction from hernias 3 (4.30%)  Complicated diverticulitis 6 (8.60%)  Perfurated viscus 8 (11.40%)  Small bowel obstuction from cancer 7 (10.00%)  Anastomotic leak 8 (11.40%)  Others 20 (28.60%) |
| Oishi, 2025 | Contaminated 22 (33.80)  Dirty 43 (66.20) | Contaminated 18 (29.00)  Dirty 44 (71.00) | GI perforations | GI perforations |
| Philip, 2024 | Clean 6 (12.80)  Clean-contaminated 26 (55.30)  Contaminated 5 (10.60)  Dirty 10 (21.30) | Clean 1 (2.04)  Clean-contaminated 25 (51.02)  Contaminated 4 (8.16)  Dirty 19 (38.78) | Benign obstruction 8( 17.00)  Benign perforation 12 (25.50)  Malignant obstruction 17 (36.20)  Malignant perforation 1 (2.10),  Ischemia 5 (10.60)  Trauma 3 (6.40)  Bleeding 1 (2.10) | Benign obstruction 6 (12.24)  benign perforation 16 (32.66)  malign obstruction 18 (36.74)  malign perforation 4 (8.16)  ischemia 2 (4.08)  trauma 1 (2.04)  bleeding 2 (4.08) |
| Rafaqat, 2024 | Clean 29  Clean-contaminated 81  Contaminated 94  Dirty/infected 183 | Clean 33  Clean-contaminated 86  Contaminated 71  Dirty/infected 197 | NR | NR |
| Reese, 2025 | Clean-contaminated 17  Contaminated 132 | Clean-contaminated 20  Contaminated 91 | NR | NR |
| Schurtz, 2018 | Clean 11  Clean-contaminated 19  Contaminated 18 | Clean 14  Clean-contaminated 20  Contaminated 14 | Bowel ischemia 7 (15)  Bowel obstruction 18 (38)  Cholecystitis 6 (13)  Perforated viscus 4 (8)  Trauma 2 (4)  Other indications 6 (12) | Bowel ischemia 4 (8)  Bowel obstruction 18 (38)  Cholecystitis 4 (8)  Perforated viscus 13 (27)  Trauma 4 (8)  Other indications 5 (10) |
| Tsukazaki, 2024 | Dirty | Dirty | Large bowel 17  - Perforated colorectal cancer 3 (13.60)  - Diverticular perforation 9 (40.90)  - Anastomotic leakage 5 (22.70)  Small bowel 5  - Iatrogenic 3 (13.60)  - Reason unknown 2 (9.10) | Large bowel 54  - Perforated colorectal cancer 6 (9.20)  - Diverticular perforation 26 (40.00)  - Anastomotic leakage 15 (23.10)  - Others 7 (10.80)  Small bowel 11  - Iatrogenic 1 (1.50)  - Foreign body 2 (3.10)  - Reason unknown 7 (10.80)  - Others 1 (1.50) |

**Supplementary Figure 1.** Forest plot comparing NPWT and standard dressing for hematoma

**Supplementary Figure 2.** Forest plot comparing iNPWT and standard dressing for postoperative complications according to the Clavien–Dindo classification, including overall complications and subgroup analyses by complication severity (Clavien–Dindo ≥ III and < III)

**Supplementary Figure 3.** Forest plot comparing NPWT and standard dressing for mortality in the subgroup analysis restricted to RCTs

**Supplementary Figure 4.** Forest plot comparing NPWT and standard dressing for 30-day readmission

**Supplementary Figure 5.** Risk-of-Bias for RCTs performed by Rob-2

**Supplementary Figure 6.** Risk-of-Bias for observational studies by Robins-I

**Supplementary Figure 7.** Leave-one-out sensitivity analysis for SSI

**Supplementary Figure 8.** Leave-one-out sensitivity analysis for SSI in the subgroup analysis restricted to RCTs

**Supplementary Figure 9.** Leave-one-out sensitivity analysis for length of hospital stay

**Supplementary Figure 10.** Leave-one-out sensitivity analysis for length of hospital stay in the subgroup analysis restricted to RCTs

**Supplementary Figure 11.** Leave-one-out sensitivity analysis for wound dehiscence in the subgroup analysis restricted to RCTs

**Supplementary Figure 12.** Baujat plot for SSI

**Supplementary Figure 13.** Baujat plot for SSI in the subgroup analysis restricted to RCTs

**Supplementary Figure 14.** Baujat plot for length of hospital stay

**Supplementary Figure 15.** Baujat plot for length of hospital stay in the subgroup analysis restricted to RCTs

**Supplementary Figure 16.** Baujat plot for wound dehiscence in the subgroup analysis restricted to RCTs

**Supplementary Figure 17.** Funnel plot for SSI

**Supplementary Figure 18.** Funnel plot for SSI in the subgroup analysis restricted to RCTs

**Supplementary Figure 19.** Funnel plot for superficial SSI

**Supplementary Figure 20.** Funnel plot for deep SSI

**Supplementary Figure 21.** Funnel plot for organ/space SSI

**Supplementary Figure 22.** Funnel plot for length of hospital stay

**Supplementary Figure 23.** Funnel plot for length of hospital stay in the subgroup analysis restricted to RCTs

**Supplementary Figure 24.** Funnel plot for ICU length of stay

**Supplementary Figure 25.** Funnel plot for wound dehiscence

**Supplementary Figure 26.** Funnel plot for wound dehiscence in the subgroup analysis restricted to RCTs

**Supplementary Figure 27.** Funnel plot for seroma

**Supplementary Figure 28.** Funnel plot for seroma in the subgroup analysis restricted to RCTs

**Supplementary Figure 29.** Funnel plot for hematoma

**Supplementary Figure 30.** Funnel plot for Clavien-Dindo classification

**Supplementary Figure 31.** Funnel plot for mortality

**Supplementary Figure 32.** Funnel plot for mortality in the subgroup analysis restricted to RCTs

**Supplementary Figure 33.** Funnel plot for 30-day readmission

**Supplementary Figure 34.** Trial sequential analysis comparing NPWT with standard dressing for SSI
